# Genetic Inheritance of Stripe Rust (*Puccinia Striiformis*) Resistance in Bread Wheat Breeding Lines at Seedling and Maturity Stages

**DOI:** 10.3390/plants11131701

**Published:** 2022-06-27

**Authors:** Saira Saleem, Muhammad Kashif, Rizwana Maqbool, Nisar Ahmed, Rubina Arshad

**Affiliations:** 1 Department of Plant Breeding and Genetics, University of Agriculture, Faisalabad 38000, Pakistan; mkashifpbg@gmail.com (M.K.); rizwana.maqbool@uaf.edu.pk (R.M.); 2 Centre of Agricultural Biochemistry and Biotechnology (CABB), University of Agriculture Faisalabad, Faisalabad 38000, Pakistan; drmiannisar@yahoo.com; 3 Plant Breeding and Genetics Division, Nuclear Institute for Agriculture and Biology, Faisalabad 38950, Pakistan; arshadrubina@hotmail.com

**Keywords:** cluster analysis, genetic studies, *Triticum aestivum*, yellow rust

## Abstract

One hundred and five (105) bread wheat (*Triticum aestivum* L.) genotypes, including five commercial checks, were screened for stripe rust resistance at seedling and adult plant stages. Seedlings grown under controlled conditions were screened for disease resistance after 12 days concerning disease incidence percentage after inoculation. K-means cluster analysis divided the genotypes into five different classes according to the presence of virulence/avirulence profile, i.e., class 1, 2, 3, 4 and 5. The same set of genotypes was grown under field conditions for adult plant resistance. Data for disease scoring and different yield and yield-related parameters was recorded. A comparison of breeding lines indicated that all studied traits were negatively affected by disease incidence. Further cluster analysis ranked the genotypes into three distinct groups with Group I and III being the most diverse. Thirteen stripe rust resistance lines were identified using seedling and adult plant resistance strategies. Correlation analysis indicated a negative association between stripe rust incidence and yield and yield-related traits, particularly grains per spike, grain weight per spike, thousand-grain weight, and grain yield per plant. These findings suggested that stripe rust resistance negatively affects yield and yield related traits. The breeding programs aiming at the development of high yielding varieties must also focus on stripe rust resistance.

## 1. Introduction

Bread wheat (*Triticum aestivum* L.), a member of the Graminae family, is a self-pollinated crop. It is grown in irrigated and rainfed areas. It is a staple crop for the whole community and that is why it is acknowledged as the “King of cereals”. Pakistan is the sixth-largest consumer of wheat worldwide [1]. Wheat is known as a reliable and inexpensive source of fibre, proteins, vitamins and minerals [2]. Their quality and quantity largely depend upon the genotype, environment, and their mutual interaction [3]. Wheat accounts for 90% of production and it contributes the maximum volume of trade worldwide. During 2019–2020, Pakistan produced 24.946 million tons of wheat grain, with an average yield of 2827 kg ha-1 [4].

Wheat (*Triticum aestivum* L.) is at stake due to the stresses, i.e., biotic and abiotic, which contribute enormously to its production losses [5,6,7]. Pertaining to biotic stress, nearly 50 diseases of wheat resulted in yield failure and amongst all fungal pathogens, rusts are of great importance. Three types of wheat rusts, i.e., stripe rusts, leaf rusts and stem rusts are significantly damaging grain yield all over the world [8,9]. In Pakistan, a drastic stripe rust epidemic occurred during 2005 that wiped out nearly all the local cultivars of wheat. Stripe rusts are the most important yield limiting factor in many parts of the world, including North America and South Asia [10,11]. In the current era, accomplishing durable resistance through the integration of several adult plant resistance genes (Yr5, Yr10, Yr15, and Yr1) are the foremost intentions [12,13]. The disease severity % and disease incidence % of yellow rust may differ region-wise all over the world because the fungus strains for rust are specified for the specific areas, i.e., *Puccinia striiformis* f. sp. hordei can cause disease [9,14,15,16,17]. However, the yield increase in wheat is not rapid enough to fulfil the food necessities of the world’s growing population by 2050 [5].

Several approaches exist to handle the stripe rust disease worldwide. The use of different agronomic practices and spraying of chemicals have proved productive to reduce the yield losses. However, the curative actions of control through chemicals are not acceptable in advanced nations. Hence, cultivation of the resistant cultivars may be a viable and inexpensive way as the environmental and health hazards could be minimal [15,18]. Evaluation of promising strains of wheat is necessary to select and identify the useful germplasm in any breeding programme. Different cultural practices, i.e., do away with alternate host plants, alternating planting dates, developing early maturing cultivars, and the use of varietal mixes are applied to avoid or minimize rust attack losses. All these practices are operative to reduce the disease susceptibility and inoculum level [19]. One of the environmentally friendly, effective, and efficient processes to avoid yield reduction is the development of resistant or moderately resistant wheat cultivars [5]. Developing the resistant cultivars involves the existence of diversity in the existing wheat germplasm against various rust pathotypes [12]. Currently, more than 50 (Yr) genes for stripe rust resistance have been acknowledged. Most of them state race-specific resistance in a gene-for-gene manner [20].

So, keeping in view the above scenario for stripe rust, the present study is performed to check the response of bread wheat genotypes against stripe rust, to assess the inheritance pattern and to identify the stripe rust resistance source.

## 2. Materials and Methods

### 2.1. Genetic Variability in Bread Wheat Genotypes

This experiment comprised of one hundred and five (105) wheat genotypes/lines, including five (5) check varieties collected from Wheat Research Institute (WRI), Ayub Agricultural Research Institute (AARI), Faisalabad, Pakistan and Department of Plant Breeding and Genetics (PBG), University of Agriculture, Faisalabad based on susceptibility and resistance for yellow rust. These genotypes/entries also carried genetic variability for grain yield and some of its related traits. Wheat genotypes were sown during the season 2015–2016. The screening was performed in two phases:

i. Seedling resistance in greenhouse conditions (SR).
ii. Adult plant resistance under field conditions (APR).

(i) Seedling Resistance:

One hundred and five (105) wheat diverse genotypes/lines of wheat, including five (5) checks, were sown under controlled conditions (Temperature = 15 ± 2 °C and relative humidity = 85%) in polythene bags at Seed Science & Technology Laboratory, University of Agriculture Faisalabad. Twelve days seedlings were inoculated with rust spores to develop the rust infestation. Inoculation and disease criteria were identical to Adult Plant Resistance. Data for disease incidence (%) was recorded on 3rd day after inoculation with the inoculum of stripe rust obtained from Plant Pathology section of Nuclear Institute for Agriculture and Biology (NIAB), Faisalabad.

(ii) Adult plant Resistance:

One hundred and five (105) wheat diverse genotypes/lines of hexaploid wheat were sown in Augmented design (non-replicated) [21] in the research area of PBG Department. One row of most susceptible check Morocco was planted after two test entries and the experiment was inoculated to develop the rust infestation at tillering stage. Any sort of fungicide/chemical spray was restricted. The following data were recoded at three different times:

1. Data for disease (stripe rust) was recorded according to the disease score developed at Plant Breeding Institute (PBI), Sydney, Australia [22].
2. Data for yield and yield related parameters.

### 2.2. Disease Scoring

(i) Inoculum and spore preparation and inoculation:

Step of Inoculum preparation, suspension, concentration and spraying on the nursery material and creation of favourable conditions for disease development were completed. After collection of fresh uredospores, suspension of rust was prepared having 250 mg urediniospores per/litre (of distilled water) and two drops of Tween-20 were also added. Using a hand sprayer, the inoculum with a pressure of 1.1 kg/cm^2^ was applied to experimental plots [23]. For each accession of the stripe rust screening plot, a border of susceptible check ‘Morocco’ was made and the experimental plots were inoculated at tillering stage (end of February) by making uniform spray of stripe rust uredospore’s suspension. The inoculum consisted of mixture of uredospores of different stripe rust strains/races. Infection and Infection category were monitored. Inoculum was applied and disease scoring [22] was performed according to Table 1. At maturity, selection of 10 random plants was made from each row and replication and data of following yield and yield related parameters, except flag leaf area, were recorded: i.e., plant height (cm), peduncle length (cm), flag leaf area (cm^2^), fertile tillers per plant, spike length (cm), number of spikelets per spike, number of grains per spike, 1000 grain weight (g), grain weight per spike (g) and grain yield per plant (g).

### 2.3. Statistical Analysis

The mean of the collected observations was taken and then examined using the software, i.e., XLstat and Statistix 9.1. Cluster analysis was used [24] to categorize the diverse genotypes into different groups. The average values of each attribute were made uniform before analysis to minimize the effect of variance in scale. Different clusters based on the genetic variability obtained, consisting of diverse and extreme genotypes, were added to the selection for the next experimental studies. Correlation examination was executed by the statistical technique defined by [25].

## 3. Results

The study aimed to identify the disease-resistant wheat genotypes through cluster analysis, which facilitated our categorization of the available germplasm into different clusters based on their genetic potential. One hundred and five (105) wheat genotypes, including five commercial checks were screened for seedling as well as adult plant resistance to check the genetic variability and disease resistance potential under the disease condition.

### 3.1. Seedling Resistance

**Figure 1:**
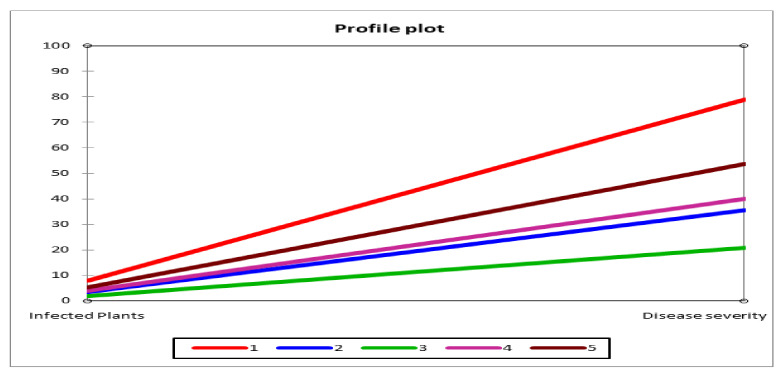
K-means cluster of 105 wheat genotypes (including 5 check varieties) based on disease incidence under disease condition at seedling stage.

Response of wheat genotypes against inoculation rust infestations for stripe rust was observed. Ten (10) seedlings of each genotype were sown in polythene bags under controlled conditions, i.e., temperature and humidity (%). The disease incidence at the seedling stage was observed by counting the number of plants on which the disease occurred. The plants were inoculated with the yellow rust inoculum two days before the data collection. The data collected for number of plants infected was analysed using the ‘SPSS’ v 12.0 for windows software. K-means cluster was constructed representing the different classes for disease incidence, as shown in Figure 1 and Table 2.

### 3.2. Adult Plant Resistance

Adult plant response of wheat genotypes against stripe rust infestations was observed at three different times using the scale (Table 1) developed at Plant Breeding Institute (PBI), Sydney, Australia. The reaction at different times was used to designate a score to that genotype.

### 3.3. Significance and Ranking

Among genotypes, highly significant variation was found for all the traits viz., plant height, flag leaf area, fertile tillers per plant, peduncle length, spike length, spikelets per spike, grains per spike, grain yield per spike, 1000-grain weight and grain yield per plant, as given in Table 3. The variation observed in blocks on all the studied traits was significant, indicating that block is effective in the case of augmented design because it is a non-replicated design. The information obtained from this study will be useful for breeding rust resistance in wheat genotypes and will provide the basis for planning breeding strategies.

The genotypes were ranked in different groups via phenograms (cluster analysis) to support the grouping of the 105 wheat genotypes (including checks) for disease resistance. The ranking was performed based on summation, i.e., the smallest and largest groups were ranked as resistant and susceptible, respectively. So, thirteen genotypes (nine resistant and five susceptible) were selected for crossing. All the traits were analysed by cluster analysis with the help of ‘SPSS’ v 12.0.

### 3.4. Cluster Analysis Based on Nine Morphological Characters

**Figure 2:**
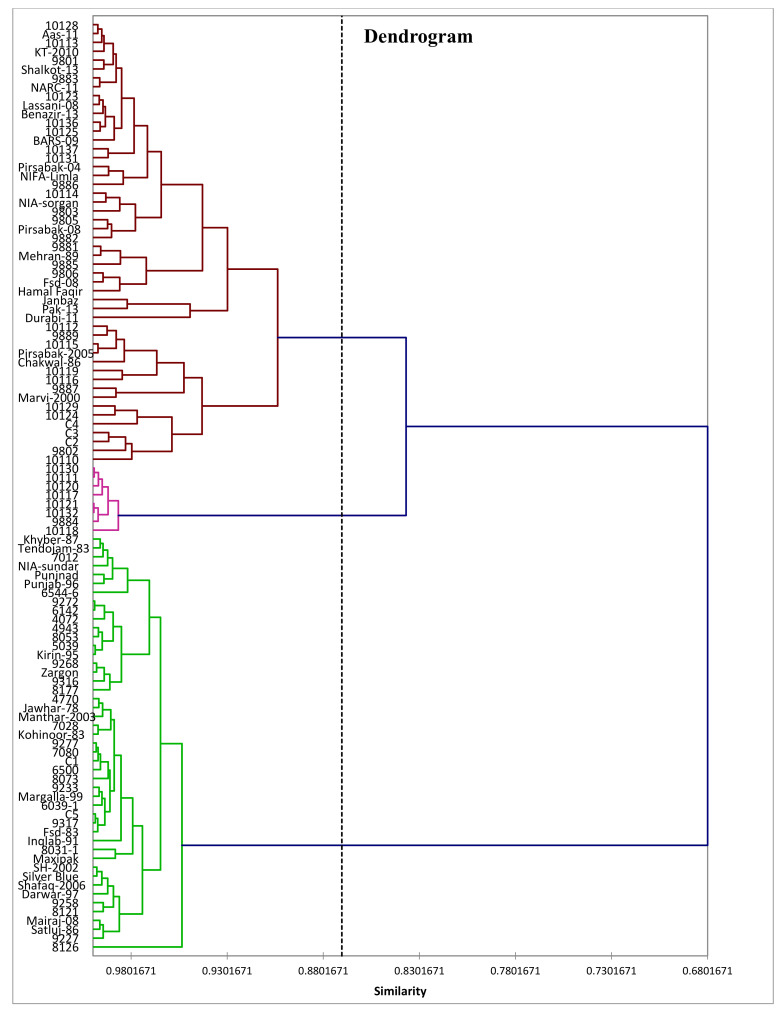
Dendrogram of 105 wheat genotypes (including 5 control (check varieties)) based on yield-related traits at maturity stage under disease condition.

The results exposed substantial phenotypical diversity existing among the material. Group association via cluster analysis for disease resistance, yield, and yield related parameters is presented in Figure 2 and Table 3. Genotypic comparison for the studied characters represented that they were significantly affected by disease, although the degree of effect varied from genotype to genotype. The final classification of the lines based on clusters constructed three groups (Table 4 and Table 5).

(I) Group-I:

In group I, 47 genotypes were placed, which were 45% of the total genotypes, indicating group I did not perform well for all the traits, i.e., disease incidence (72%), plant height (105.02), flag leaf area (23.83), fertile tillers per plant (3.00), peduncle length (9.762), spike length (9.933), spikelets per spike (21.540), number of grains per spike (34.043), grain weight per spike (1.180), grain weight per plant (10.769) and 1000 grain weight (34.878).

(II) Group-II:

In group II, 50 genotypes were placed, which were 47% of the total genotypes. The performance of different traits in this group is as follows: disease incidence % (32.580), plant height (83.35), flag leaf area (34.09), fertile tillers per plant (15.90), peduncle length (10.866), spike length (11.088), spikelets per spike (22.35), grains per spike (41.212), grain weight per spike (1.587), grains weight per plant (12.219) and 1000 grain weight (39.024).

(III) Group-III:

Group III included the remaining eight genotypes, which were 8% of the total genotypes. So, this group performed best, indicating resistance to stripe rust (minimum disease incidence 20%) and also showed significant good performance for the traits, i.e., plant height (51.09), flag leaf area (44.90), fertile tillers per plant (9.50), peduncle length (12.500), spike length (13.863), spikelets per spike (23.250), grains per spike (65.875), grain weight per spike (2.775), grain weight per plant (25.328) and 1000 grain weight (50.318).

Correlation values for stripe rust infestation were observed to be negatively significant towards most of the yield contributing traits, such as spike length, number of grains per spike, grain weight per spike, grain weight per plant and 1000 grain weight signifying the importance of these traits against stripe rust. Although, some other plant traits, such as fertile tillers per plant, plant height, flag leaf area, length of peduncle and spikelets/spike were not affected by yellow rust because the non-significant value of the correlation coefficient was observed for these traits (as described in Table 6).

## 4. Discussion

Effective implementation of any breeding programme is mainly dependent upon the choice of the most diverse and promising gene pool, keeping in view the main target, i.e., high yield for any breeding program is only possible by the accumulation of possible desirable traits into a single cultivar. It could be achieved by identifying the material having desirable qualities and then hybridizing it to accumulate maximum desirable traits into a particular cultivar [26]. Cluster analysis at seedling stage and maturity was used to determine the magnitude of divergence in the genotypes and to identify the desired genotypes for exploitation in the hybridization programme [27,28,29]. In the current research, 105 wheat genotypes were screened at seedling as well as maturity stage using cluster analysis. Testing at the seedling stage divided genotypes into five different groups using K-means cluster analysis, which revealed the presence of virulence/avirulence profile, i.e., class 1, 2, 3, 4, and 5 included 42, 9, 36, 10, and 8 genotypes, respectively [30,31,32]. The varieties in this research performing well have been reported to show resistance under field conditions also in many regions of Pakistan. The majority of remaining cultivars and lines lacked seedling resistance [33,34,35].

At maturity, the analysis showed highly significant variances among the genotypes for all traits under study. The genotypes that were resistant or moderately resistant had also shown good mean values for yield and its related traits whereas the susceptible ones had shown the minimum yield. The genotypes grouped into three groups exhibited maximum genetic divergence, indicating that the group having the highest trait mean for disease incidence and lowest trait mean is considered as susceptible. On the other hand, the genotypes having the lowest mean for disease incidence and highest mean for yield and its related traits were considered as resistant genotypes. Similar findings were also reported by [26,36,37]. The genotypes presented in groups will be more diversified and could be utilized in the hybridization programme for developing high yielding varieties under disease situations [38,39].

Outcomes showed that genotypes selected from the experiment at the seedling stage also performed similarly at the maturity stage, suggesting that wheat genotypes at the seedling stage can be screened for stripe rust resistance [40,41,42,43,44]. Genotypes with consistent performance under disease conditions at seedling as well as maturity stage were selected to be used in the hybridization program to generate the genetic material for further studies. The crosses produced from genotypes within compatibility limits of clusters might yield appropriate transgressive segregants. This might be helpful in breeding high yielding varieties. Adult plant response (APR) to stripe rust was scaled and the association between yield and disease was discovered by correlation coefficient values and it was detected that yellow rust infestation had a highly significant positive value of association for the disease incidence, indicating that the higher the disease incidence, the greater the disease severity will be. It is concluded that the genotypes with the low stripe rust infestation and the higher value of yield and yield related parameters could be selected to develop the desirable stripe rust resistant cultivars.

### Future Recommendations

1. Wheat genetic resources for stripe rust resistance must be considered in parts of the country with extreme weather conditions (high temperature) to investigate their reliability.
2. Diverse foundations of resistance, i.e., seedling and adult plant resistance in acknowledged germplasm, may be utilized to study the genetics of resistance. Observation of the stripe rust virulence pattern ought to be carried out frequently.
3. Utilization of the studied germplasm will be valuable in future wheat breeding programs.

## Figures and Tables

**Table 1:** Major infection type classes for stripe rust (McIntosh, 1995).

Infection Type	Host Response	Symptoms	Adult Plant Response	Codes
0	Immune	No visible Uredia	Resistant (R)	1
N	Very resistant	Hypersensitive flecks		
1	Resistant	Small Uredia with necrosis		
2	Resistant to moderately resistant	Small to medium size Uredia with green islands and surrounded by necrosis and chlorosis	Moderately resistant (MR)	2
3	Moderately resistant to moderately susceptible	Medium size Uredia with and without chlorosis	Moderately Susceptible (MS)	3
4	Moderately susceptible	Large Uredia with chlorosis	Susceptible (S)	4

**N:** indicated more than usual degree of necrosis; **(fleck)**: indicated more than usual degree of chlorosis.

**Table 2:** Response of wheat genotypes based on disease incidence (DI %).

Class	No. of Genotypes	Genotypes
1	42	Silver Blue, Zargon, Inqlab-91, Kohinoor-83, Maxipak, Punjab-96, Tendojam-83, SH-2002, Margalla-99, Manthar-2003, Khyber-87, Punjnad, Darwar-97, Shafaq-2006, Satluj-86, Mairaj-08, Jawhar-78, NIA-sundar, Fsd-83, Kirin-95, 6039-1, 6142, 6500, 6544-6, 7012, 7028, 7080, 8031-1, 8053, 8073, 8126, 8177, 4072, 4770, 4943, 9258, 9268, 9277, 9316, 9317, C1 and C5
2	9	Chakwal-86, Marvi-2000, Lasani-08, Fsd-08, NARC-11, 9887, 10119, C3 and C4
3	36	Benazir-13, Mehran-89, Pirsabak-2005, Aas-11, BARS-09, Pirsabak-08, Janbaz, NIFA-Limla, KT-2010, Sialkot-13, 10114,10118, 10123, 10125, 10128, 10131, 10132, 10136, 10137, 9805, 9882, 9883, 9884, 9886, 9801, 9889, 10111, 10112, 10115, 10116, 10117, 10120, 10121, 10124, 10129 and 10130
4	10	Durabi-11, Pak-13, Hamal Faqir, NIA-sorgan, Pirsabak-04, 10113, 9802, 9803, 9885 and 10110
5	8	9806, 9881, 5039, 8121, 9227, 9233, 9272 and C2

**Table 3:** Means for disease incidence (%) and yield parameters.

Sr. No.	Trait	Cluster I	Cluster II	Cluster III
1	Disease incidence (%)	72.07	32.58	20.00
2	Plant height (cm)	105.02	83.35	51.09
3	Flag leaf area (cm^2^)	23.83	34.09	44.90
4	Fertile tillers per plant	3.00	15.9	9.50
5	Peduncle Length (cm)	9.76	10.87	12.50
6	Spike Length (cm)	9.93	11.09	13.86
7	Spikelets per Spike	21.54	22.35	23.25
8	Grains per spike	34.04	41.21	65.88
9	Grain weight per spike (g)	1.18	1.59	2.78
10	Grain yield per plant (g)	10.77	12.22	25.24
11	1000 Grain weight (g)	34.88	39.02	50.32

**Table 4:** Analysis of variance for yield and yield-related traits in wheat.

SOV	d.f	Plant Height	Flag Leaf Area	Peduncle Length	Fertile Tillers Per Plant	Spike Length	Number of Spikelets Per Spike	Number of Grains Per Spike	1000 Grain Weight	Grain Weight Per Spike	Grain Yield Per Plant
**Blocks**	4	24.46 *	22.84 *	17.77 *	40.23 *	1.38 *	9.96 *	60.86 *	6.59 *	1.12 *	8.19 *
**Genotypes**	4	81.66 *	44.94 *	6.68 *	22.22 *	5.37 *	8.96 *	577.46 *	45.92 *	6.32 *	52.16 *
**Error**	16	5.03	6.54	5.02	6.35	0.60	2.55	12.96	2.52	0.40	2.62

* = Significant *p* ≤ 0.05.

**Table 5:** Screening results of adult plant resistance (under field condition) experimental design.

Class No.	No. of Genotypes	Genotype Name	Significant Traits
1	47	Khyber-87, Tendojam-83, 7012, NIA-sundar, Punjnad, Punjab-96, 6544-6, 9272, 6142, 4072, 4943, 8053, 5039, Kirin-95, 9268, Zargon, 9316, 8177, 4770, Jawhar-78, Manthar-2003, 7028, Kohinoor-83, 9277, 7080, C1, 6500, 8073, 9233, Margalla-99, 6039-1, C5, 9317, Fsd-83, Inqlab-91, 8031-1, Maxipak, SH-2002, Silver Blue, Shafaq-2006, Darwar-97, 9258, 8121, Mairaj-08, Satluj-86, 9227, 8126	**Highest traits (means)** Disease incidence **Average traits (means)** **N/A** **Lowest traits (means)** Number of grains per spike, gain yield per plant, 1000 grain weight, spike length, spikelets per spike, grain weight per spike and peduncle length
2	50	10128, Aas-11, 10113, KT-2010, 9801, Sialkot-13, 9883, NARC-11, 10123, Lasani-08, Benazir-13, 10136, 10125, BARS-09, 10137, 10131, Pirsabak-04, NIFA-lima, 9886, 10114, NIA-sorgan, 9803, 9805, Pirsabak-08, 9882, 9881, Mehran-89, 9885, 9806, Fsd-08, Hamal Faqir, Janbaz, Pak-13, Durabi-11, 10112, 9889, 10115, Pirsabak-2005, Chakwal-86, 10119, 10116, 9887, Marvi-2000, 10129, 10124, C44, C3, C2, 9802, 10110	**Highest traits (means)** N/A **Average traits (means)** Disease incidence, spike length, number of grains per spike, grain weight per plant, 1000 grain weight, spikelets per spike, grain weight per spike and peduncle length **Lowest traits (means)** **N/A**
3	8	10130, 10111, 10120, 10117, 10121, 10132, 9884, 10118	**Highest traits (means)** Spike length, spikelets per spike, number of grains per spike, grain weight per spike, grain weight per plant and 1000 grains weight **Average traits (means)** N/A **Lowest traits (means)** Peduncle length and disease incidence

**Table 6:** Correlation coefficient values of stripe rust with different yield contributing traits.

Sr. No.	Trait	Correlation Value for Stripe Rust
1	Disease incidence (%)	0.870 **
2	Plant height (cm)	−0.070 ^N.S^
3	Flag leaf area (cm^2^)	0.117 ^N.S^
4	Fertile tillers per plant	0.074 ^N.S^
5	Peduncle Length (cm)	0.162 ^N.S^
6	Spike Length (cm)	−0.217 *
7	Spikelets per Spike	0.007 ^N.S^
8	Grains per spike	−0.680 **
9	Grain weight per spike (gm)	−0.740 **
10	Grain yield per plant (gm)	−0.524 **
11	1000 Grain weight (gm)	−0.506 **

** = Highly Significant *p* ≤ 0.01, * = Significant *p* ≤ 0.05, ^N.S^ = Non significant.
